# Development of HPMC-Based Hard Capsules with Rapid Disintegration Across Simulated Gastrointestinal pH Conditions: Formulation Design, Process Optimization, and Disintegration Mechanism of the HPMC/GG/ι-C Ternary System

**DOI:** 10.3390/md24050162

**Published:** 2026-05-02

**Authors:** Yuting Dong, Songlin Ye, Xiaojun Hong, Yafang Shi, Youcheng Liu, Xueqin Zhang, Jing Ye, Meitian Xiao

**Affiliations:** 1 College of Chemical Engineering, Huaqiao University, Xiamen 361021, China; 23011087007@stu.hqu.edu.cn (Y.D.); xqzhang2009@hqu.edu.cn (X.Z.); 2 Anhui Huangshan Capsule Co., Ltd., Xuancheng 242600, China; ysl@hsjn.com (S.Y.);; 3 Fujian Green Power Biotechnology Co., Ltd., Zhangzhou 363100, China; unhong0328@163.com; 4 Instrumental Analysis Center of Huaqiao University, Xiamen 361021, China; shiyafang1997@163.com

**Keywords:** hydroxypropyl methylcellulose, gellan gum, ι-carrageenan, hard capsules

## Abstract

While hydroxypropyl methylcellulose (HPMC) is a promising plant-based alternative to gelatin, its industrial application is limited by poor mechanical properties and high production costs. In this study, high-performance HPMC-based hard capsules were developed using an HPMC/gellan gum/ι-carrageenan ternary system. The formulation and preparation process were optimized via single-factor experiments, response surface methodology, and low-field nuclear magnetic resonance analysis. Scanning electron microscopy was applied to characterize the microstructural evolution during disintegration. The optimized capsules exhibited rapid disintegration within 15 min across four pH media and satisfied the requirements of the Chinese Pharmacopoeia (2025). Drug dissolution profiles using cefradine and ranitidine hydrochloride showed over 85% cumulative release within 30 min, with similarity factors higher than 50 relative to commercial gelatin capsules under the tested conditions. This work provides a feasible and low-cost strategy for the industrial production of plant-based capsules and promotes the high-value utilization of polysaccharide-based capsule materials.

## 1. Introduction

Oral solid dosage forms represent one of the most widely administered formulations in clinical practice, with hard capsules serving as essential carriers that directly influence drug release behavior and bioavailability [1]. Traditional gelatin capsules have long dominated the market due to their favorable film-forming properties and biocompatibility [2]. However, they exhibit inherent limitations, including animal-derived safety risks (e.g., potential transmission of bovine spongiform encephalopathy and foot-and-mouth disease), susceptibility to cross-linking with certain drugs leading to delayed dissolution, poor moisture stability, and incompatibility with religious and vegetarian dietary restrictions [2,3]. These drawbacks have constrained their further application in modern pharmaceutical development. Consequently, the development of plant-based capsule materials derived from natural sources, possessing stable performance and adaptability to diverse drug delivery scenarios, has emerged as a critical research focus and core requirement in the field of pharmaceutical excipients [4].

Hydroxypropyl methylcellulose (HPMC), a Generally Recognized as Safe (GRAS) food-grade polymer, exhibits excellent film-forming properties, biocompatibility, chemical inertness, and low hygroscopicity [5]. These features make it an attractive plant-based alternative to gelatin. However, capsules prepared solely from HPMC suffer from insufficient mechanical strength and limited controllability over disintegration behavior, particularly struggling to achieve rapid disintegration and synchronized drug release within the complex pH environment of the gastrointestinal tract [6,7]. Moreover, the traditional thermal gelation process for HPMC capsules is complicated, energy-intensive, and highly dependent on equipment precision, which hinders industrial scale-up [8]. Therefore, developing HPMC-based capsules with improved mechanical properties, stable processability, and reliable rapid disintegration remains a major challenge in the field of pharmaceutical excipients.

An effective strategy to address these limitations involves blending HPMC with natural polysaccharide gelling agents, leveraging synergistic interactions between different polysaccharides to optimize the structural and functional properties of the capsule film [9]. Low-acyl gellan gum (GG) is a linear anionic polysaccharide produced by fermentation from Sphingomonas elodea [10]. Its molecular chain is composed of glucose, glucuronic acid, and rhamnose in a specific repeating ratio, conferring advantages such as high gel strength, uniform film-forming ability, good thermal stability, and biodegradability [11]. Accordingly, GG has been widely integrated into the development of pharmaceutical hard capsules. By synergistically forming a three-dimensional gel network with film-forming agents, GG significantly enhances the mechanical strength and dimensional stability of capsules, preventing issues such as brittleness and powder leakage during manufacturing. For instance, Fülöpová et al. prepared capsules by blending HPMC with GG to achieve small intestine administration [12]. However, in the context of fast-disintegrating capsule applications, GG exhibits notable performance limitations. In acidic environments (e.g., simulated gastric fluid at pH 1.2), the carboxyl groups on its molecular chains undergo protonation, leading to reduced hydrophilicity. This protonation induces densification of the gel network, hindering rapid water penetration and consequently resulting in delayed disintegration [13]. Furthermore, in neutral to weakly alkaline environments, the crosslinking interactions of GG remain excessively strong, similarly impeding rapid swelling and structural dissociation of the film [14]. These characteristics render GG alone unable to meet the core requirement of rapid disintegration essential for oral solid dosage forms.

In contrast, ι-carrageenan (ι-C), a marine-derived sulfated polysaccharide extracted from red algae [15], offers a distinct pharmaceutical advantage that differs from non-marine gelling agents such as GG. Owing to the abundant sulfate ester groups on its molecular chains, ι-C exhibits strong hydrophilicity and characteristic pH-responsive hydration behavior [16]. In acidic environments relevant to gastric conditions, these sulfate groups help maintain electrostatic repulsion and water accessibility within the polymer network, thereby facilitating rapid water penetration, swelling, and matrix loosening [17]. This feature is pharmaceutically important for immediate-release capsule systems, in which prompt capsule opening and consistent drug release depend strongly on fast hydration and disintegration. However, gel networks formed solely by ι-C generally show insufficient mechanical strength and elasticity, making the resulting capsules prone to damage during demolding, transportation, and filling [15,18]. Accordingly, in the present study, GG was used as a structural reinforcement component, while marine-derived ι-C was introduced as the key functional component to promote rapid disintegration, thereby integrating the mechanical advantages of GG with the unique pharmaceutical value of a marine sulfated polysaccharide.

Based on the complementary properties of these two polysaccharides, this study proposes blending GG and ι-C as a combined gelling agent, in conjunction with HPMC, to construct a novel plant-based capsule shell material. Key formulation variables, including total gelling-agent content, GG/ι-C ratio, coagulant composition (potassium citrate and sodium carbonate), and plasticizer type (glycerol), were systematically optimized using rheological analysis and response surface methodology. The resulting capsules were further evaluated for disintegration and drug-release performance in four simulated gastrointestinal media (pH 1.2, 4.5, 6.8, and 7.0), with the aim of assessing the feasibility of ι-C as a marine functional biomaterial for rapid-disintegrating capsule applications.

The optimized capsules met all requirements of the Chinese Pharmacopoeia (2025) and achieved rapid, consistent disintegration across the entire gastrointestinal pH range, with dissolution profiles similar to commercial gelatin capsules. This work provides a reproducible and process-feasible strategy for high-performance fast-disintegrating plant-based capsules and offers a competitive alternative to conventional HPMC and gelatin capsules.

## 2. Results

### 2.1. Formulation Screening of Capsules

#### 2.1.1. Screening of Gelling Agent Content and Ratio

**Figure 1:**
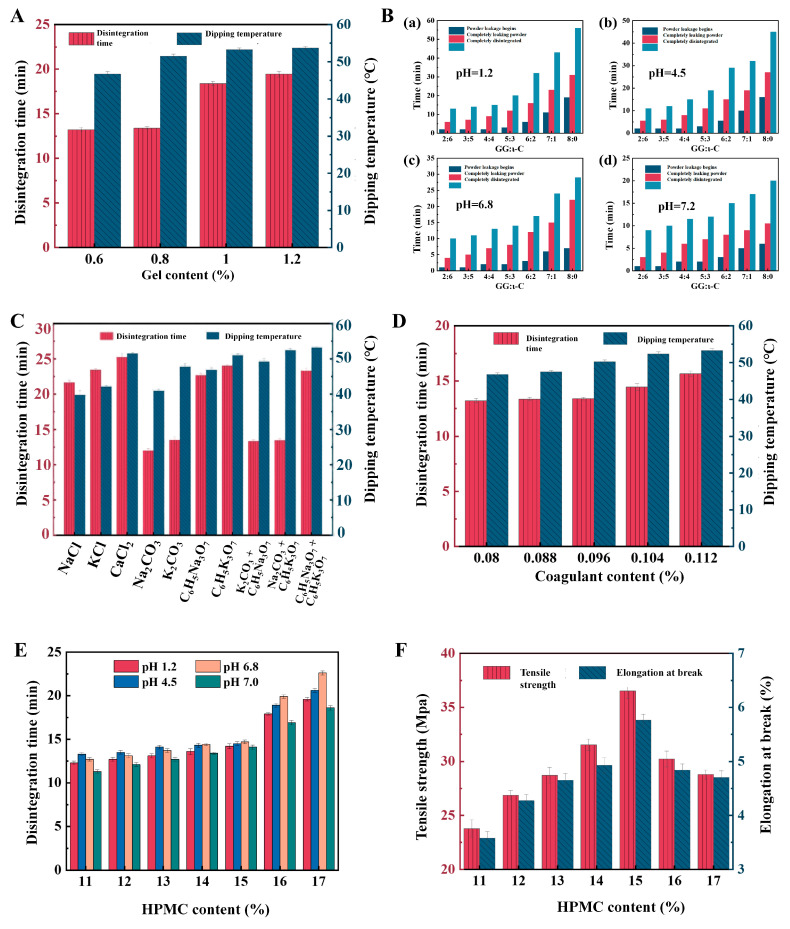
Optimization of formulation parameters for HPMC hard capsules. (**A**) Effects of total gelling agent concentration on disintegration time and dipping temperature; (**B**) influence of the GG/ι-C mass ratio on capsule disintegration; (**C**,**D**) effects of coagulant type and concentration on disintegration profiles and dipping temperature; (**E**) pH-dependent disintegration profiles of capsules with varying HPMC content across four distinct media; (**F**) mechanical properties of capsule films as a function of HPMC concentration.

Owing to the acid resistance of GG, composite polysaccharide films containing GG exhibit slow dissolution in acidic solutions. Therefore, the disintegration time in pH 1.2 medium was measured to determine the optimal total content of the gelling agents. The HPMC was fixed at 15.0% (*w*/*v*), the total content of potassium citrate and sodium carbonate was fixed at 0.10% (*w*/*v*) (mass ratio 1:1), and the mass ratio of GG to ι-C was maintained at 1:1. The effects of the total GG/ι-C content (0.60%, 0.80%, 1.00%, and 1.20% *w*/*v*) on the dipping temperature and the disintegration time in pH 1.2 medium were systematically investigated, and the results are shown in Figure 1A. It can be seen from the figure that the dipping temperature exhibited a continuous increasing trend as the total GG/ι-C content increased. When the total content was 0.60% (*w*/*v*), the dipping temperature was relatively low (approximately 45 °C). This was attributed to the insufficient gelling agent concentration, which resulted in a low cross-linking density of the three-dimensional network structure in the gum solution and a correspondingly lower energy threshold required for gelation [19]. However, the excessively low gel strength caused the capsules to be prone to deformation and breakage during the demolding and drying processes, making them difficult to meet the process requirements for industrial production. When the total content was increased to 1.00% or 1.20% (*w*/*v*), the disintegration time of the capsules in pH 1.2 medium was prolonged to 18 min and 19 min, respectively, which exceeded the requirement of complete disintegration within 15 min specified in the Chinese Pharmacopoeia (2025). In contrast, when the total GG/ι-C content was 0.80% (*w*/*v*), the disintegration time of the capsules in pH 1.2 medium was approximately 12 min. This not only satisfied the Chinese Pharmacopoeia (2025) requirement for rapid disintegration but also allowed the formation of a sufficiently strong gel network, maintaining the dipping temperature within a suitable process window (approximately 50 °C) and ensuring the molding stability of the capsules. Therefore, considering both the disintegration performance and process feasibility, the total content of GG/ι-C was determined to be 0.80% (*w*/*v*) in this study.

After determining the total gelling agent content to be 0.80% (*w*/*v*), the present study further adjusted the mass ratio of GG to ι-C to optimize the disintegration behavior of the capsules across the full range of gastrointestinal pH environments. Figure 1B characterizes the disintegration kinetics of capsules with different GG/ι-C ratios in four simulated media (pH 1.2, 4.5, 6.8, and 7.0) using three key time points: initial powder leakage, complete powder leakage, and complete dissolution. It should be noted that when the GG/ι-C ratio was 0:8 (pure ι-C), the gel strength of the solution was insufficient, leading to “slippage” during the dipping process, which prevented the formation of intact capsule shells. Therefore, this ratio was excluded from the disintegration performance evaluation. Overall, the time points of initial powder leakage, complete powder leakage, and complete dissolution of the capsules exhibited similar trends across all four pH media, indicating that the GG/ι-C ratio is a core factor regulating the disintegration behavior. Specifically, in acidic media (pH 1.2 and 4.5), the disintegration time of the capsules was significantly prolonged with an increase in GG content. This phenomenon was attributed to the protonation of carboxyl groups on the GG molecular chains under acidic environments. Protonation enhanced intermolecular hydrogen bonding and led to the formation of a denser three-dimensional network, thereby significantly delaying water penetration and the swelling and dissociation of the membrane material [20]. In contrast, the dissolution time of the capsules in acidic media was markedly shortened as the proportion of ι-C increased. The superior disintegration-related behavior observed in formulations containing ι-C may be attributed to its sulfate-rich marine polysaccharide structure, which promotes hydration and swelling more effectively than non-marine gelling agents dominated by carboxyl-mediated gelation [21]. In weakly acidic to neutral media (pH 6.8 and 7.0), the disintegration times of capsules with all ratios were generally shorter than those in acidic media, and a more rapid disintegration trend was observed with an increasing proportion of ι-C. This behavior may be attributed to the deprotonation of GG carboxyl groups under these conditions, which reduced the compactness of the gel network. At the same time, the hydrophilic advantage of ι-C became more evident, enabling the membrane to absorb water, swell, and disintegrate more rapidly [22]. By integrating the disintegration results from all four pH media, it was found that when the GG/ι-C ratios were 2:6, 3:5, and 4:4, the complete disintegration time of the capsules in all tested media was controlled within 15 min, meeting the requirements for fast-disintegrating capsules specified in the Chinese Pharmacopoeia (2025) [23].

#### 2.1.2. Selection and Determination of Coagulant Type and Content

The present study further focused on the screening and content optimization of coagulants to precisely regulate the gelation behavior of the gum solution and the disintegration performance of the capsules. As key components inducing polysaccharide cross-linking, coagulant types and dosages were found to directly affect the gel strength of the gum solution, the feasibility of the dipping process, and the disintegration characteristics of the final capsules. Under the fixed conditions of 15.00% (*w*/*v*) HPMC, 0.80% (*w*/*v*) total GG/ι-C content, a GG/ι-C mass ratio of 1:1, and a total coagulant mass fraction of 0.10% (*w*/*v*), the effects of different coagulants (NaCl, KCl, calcium chloride, sodium carbonate, potassium carbonate, sodium citrate, potassium citrate, sodium carbonate + potassium citrate, and potassium carbonate + potassium citrate) on the dipping temperature and the disintegration time of capsules in pH 1.2 medium were investigated. The results were presented in Figure 1C. Obvious differences were observed in the dipping temperatures induced by different coagulants, following the overall trend: divalent calcium salts > monovalent potassium salts > monovalent sodium salts. This phenomenon was attributed to the differential binding affinities between the carboxyl groups on GG molecular chains and cations. Divalent cations (e.g., Ca^2+^) formed stronger ionic cross-links with GG, which significantly increased the compactness of the gel network and thus elevated the temperature threshold required for gelation. Among monovalent cations, GG exhibited a higher binding affinity for K^+^ than for Na^+^, resulting in a higher gelation temperature induced by potassium salts [24]. Based on the disintegration results reported in Section 2.1.1, the capsules exhibited the longest disintegration time in pH 1.2 simulated gastric fluid, which was identified as the critical limiting condition for evaluating disintegration performance. Therefore, the disintegration behavior of capsules prepared with different coagulants was primarily investigated in pH 1.2 medium. As was shown in Figure 1C, the disintegration time of capsules was significantly shortened when sodium carbonate or potassium carbonate was used as the coagulant. Based on a comprehensive consideration of both the feasibility of the dipping process and disintegration performance, a composite coagulant system consisting of potassium citrate and sodium carbonate was selected in this study to balance gelation regulation and rapid disintegration in acidic media.

After confirming the mass ratio of potassium citrate to sodium carbonate as 1:1, the effects of total coagulant content (0.080%, 0.088%, 0.096%, 0.104%, and 0.112% *w*/*v*) on the dipping temperature and the disintegration time of capsules in pH 1.2 medium were further investigated, with the results shown in Figure 1D. At a low total coagulant content (e.g., 0.080% *w*/*v*), the dipping temperature was relatively low (approximately 48 °C). However, the excessively low gel strength rendered the capsules susceptible to deformation and breakage during demolding and drying, which failed to meet the requirements of industrial production. With the increase in coagulant content, the dipping temperature increased gradually, indicating an improvement in cross-linking density and gel strength. Nevertheless, when the content exceeded 0.104% *w*/*v*, the disintegration time of the capsules was prolonged to more than 15 min, which exceeded the requirements specified in the Chinese Pharmacopoeia (2025) [23]. This was because excessive cations induced the formation of an overly dense three-dimensional network structure, which significantly hindered water molecule penetration and the swelling-dissociation of the membrane material, leading to delayed disintegration [13]. At a total coagulant content of 0.096% *w*/*v*, the dipping temperature was 50.2 °C, which not only ensured the formation of a gel network with sufficient strength to maintain molding stability but also controlled the disintegration time of the capsules in pH 1.2 medium at 13.4 min, complying with the pharmacopoeia standards. Therefore, considering both process feasibility and disintegration performance comprehensively, the total coagulant content was determined to be 0.096% *w*/*v* in this study.

#### 2.1.3. Determination of HPMC Content

After confirming the gelling agent and coagulant systems, the present study further investigated the effects of HPMC content on the disintegration performance and mechanical strength of the capsules. In the experiments, the total content of GG/ι-C was fixed at 0.80% (*w*/*v*, mass ratio 1:1), and the total content of potassium citrate and sodium carbonate was fixed at 0.096% (*w*/*v*, mass ratio 1:1). Capsules were prepared by varying the HPMC content from 11.00% to 17.00% (*w*/*v*).

Figure 1E shows the disintegration time of capsules with different HPMC contents in four pH media. At excessive HPMC levels (>15.00%, *w*/*v*), the dense polymeric matrix hindered water permeation. This resulted in disintegration times exceeding 15 min, which failed to meet pharmacopoeial standards. In contrast, within the range of 11.00–15.00% (*w*/*v*), the capsules could completely disintegrate within 15 min in all tested media. Good mechanical properties are crucial for the industrial production and application of capsules, ensuring molding stability, preventing brittleness, and improving quality control [25]. As shown in Figure 1F, the tensile strength and elongation at break of the capsules exhibited a non-linear trend of first increasing and then decreasing with the increase in HPMC content. When the HPMC content reached 15.00% (*w*/*v*), both parameters peaked. This was attributed to the fact that an appropriate HPMC concentration promoted hydrogen bond cross-linking between molecular chains, forming a dense and flexible network structure [26]. However, when the content exceeded 15.00%, intramolecular hydrogen bonding competed more strongly, weakening intermolecular interactions and leading to a decrease in membrane strength and flexibility [27]. Based on the comprehensive results of disintegration performance and mechanical strength tests, the HPMC content was determined to be 15.00% (*w*/*v*) in this study to balance the rapid disintegration characteristics and stable process feasibility.

### 2.2. Single-Factor Experiment

**Figure 2:**
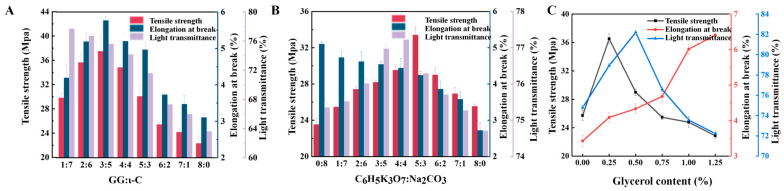
Single-factor experiments: Effects of GG/ι-C ratio (**A**), potassium citrate/sodium carbonate ratio (**B**), and glycerol content (**C**) on mechanical properties and light transmittance of capsule films.

To further optimize the formulation for subsequent response surface methodology experiments, single-factor tests were conducted to evaluate the effects of different GG/ι-C ratios on the mechanical properties and light transmittance of capsule films. With the total content of potassium citrate and sodium carbonate fixed at 0.096% (*w*/*v*) at a mass ratio of 1:1, and HPMC content fixed at 15.00% (*w*/*v*), the GG/ι-C ratio was varied while maintaining the total content at 0.80% (*w*/*v*). The prepared capsule films were then subjected to mechanical property and light transmittance testing. The effects of different GG/ι-C ratios on the mechanical properties and light transmittance of the capsule films are presented in Figure 2A. Compared to the non-blended capsule films (i.e., GG/ι-C ratios of 1:7 and 8:0), the blended GG/ι-C films exhibited superior tensile strength [28]. Light transmittance decreased with increasing GG content, which may be attributed to the relatively dense structure formed during gelation due to stronger intermolecular interactions between GG molecular chains. The structural heterogeneity within the film induces continuous light scattering and absorption during transmission, thereby reducing overall transparency. Thus, based on a holistic assessment of mechanical integrity and light transmittance, and considering the disintegration profiles detailed in Section 2.1.1, the optimal GG/ι-C mass proportions were identified as 2:6, 3:5, and 4:4. To further investigate the synergistic effects of coagulants on capsule film properties, single-factor experiments were conducted by varying the mass ratio of potassium citrate (C_6_H_5_K_3_O_7_) to sodium carbonate (Na_2_CO_3_). The total GG/ι-C content was fixed at 0.80% (*w*/*v*) with a mass ratio of 1:1, and the HPMC content was maintained at 15.00% (*w*/*v*). While keeping the total coagulant content constant at 0.096% (*w*/*v*), the potassium citrate/sodium carbonate ratio was varied. The prepared capsule films were then subjected to mechanical property and light transmittance testing. The effects of different potassium citrate/sodium carbonate ratios on the mechanical properties and light transmittance of the capsule films are presented in Figure 2B. An appropriate combination of K^+^ and Na^+^ ions was found to promote polyelectrolyte complex electrostatic interactions between the anionic polysaccharides of GG and the anionic sulfate groups of ι-C, resulting in enhanced tensile strength [29]. The elongation at break gradually decreased with increasing K^+^ content, which may be attributed to the fact that K^+^ is a typical cation that promotes GG gelation. Gels formed by GG are characterized as being more brittle and rigid, exhibiting poor flexibility, which accounts for the observed decrease in elongation at break [30]. Light transmittance reached its maximum when potassium citrate and sodium carbonate were present in comparable proportions. This phenomenon likely occurs because the combined action of both ions during the gelation process facilitates the formation of a relatively uniform and dense microstructure in the capsule film [31]. The resulting smooth film surface with fewer internal defects presents minimal obstruction to light propagation, thereby enhancing light transmittance. Based on a comprehensive evaluation of mechanical properties and light transmittance, the optimal range of potassium citrate/sodium carbonate ratios was determined to be 3:5, 4:4, and 5:3.

Hard capsules prepared from gels of natural biodegradable polymers typically exhibit inferior mechanical properties, rendering them highly susceptible to cracking and embrittlement, which limits their applicability and feasibility in production [32]. Therefore, the incorporation of plasticizers into the product formulation is necessary. Plasticizer molecules can intercalate between polymer molecular chains, weakening the intermolecular forces and increasing the degree of freedom for chain movement, thereby reducing film brittleness and enhancing flexibility [33]. In this study, glycerol was selected as the plasticizer. With the total GG/ι-C content fixed at 0.80% (*w*/*v*) at a mass ratio of 1:1, HPMC content fixed at 15.00% (*w*/*v*), and the total content of potassium citrate and sodium carbonate fixed at 0.096% (*w*/*v*) at a mass ratio of 1:1, the glycerol content was varied. The prepared capsule films were then subjected to mechanical property and light transmittance testing. As shown in Figure 2C, the tensile strength initially increased and then decreased with the addition of glycerol. This phenomenon may be related to improved dispersion of HPMC within the polymer matrix upon glycerol incorporation [34]. As a plasticizer, glycerol is a small molecular compound capable of penetrating between polymer macromolecular chains, which increases the free volume between polymer chains, thereby reducing physical entanglement and ultimately leading to a decrease in tensile strength [35]. Elongation at break represents the maximum change in sample length prior to fracture. The unplasticized composite film exhibited an elongation at break of 3.43 ± 0.16%, which increased significantly with increasing glycerol concentration. This enhancement occurs because glycerol promotes polymer chain mobility, thereby facilitating the formation of films with greater ductility and flexibility [36]. Consequently, the incorporation of an appropriate amount of glycerol can enhance mechanical properties. Light transmittance initially increased and then decreased with increasing glycerol content, indicating that excessive glycerol addition reduces the light transmittance of GG/ι-C composite films. Based on a comprehensive evaluation of mechanical properties and light transmittance, the optimal range of glycerol content was determined to be 0.25%, 0.50%, and 0.75%.

### 2.3. Response Surface Methodology

A Box–Behnken design with 17 runs (including 5 center points) was employed to optimize the formulation. The factor levels are listed in Table 1, and the experimental design with corresponding results is presented in Table 2. Quadratic polynomial regression models were established using Design-Expert software. For tensile strength (σt), the fitted model was

σt = 29.54 − 2.81A − 0.18B + 2.90C − 0.54AB + 2.51AC − 0.20BC − 2.29A^2^ − 3.44B^2^ − 2.56C^2^.

The model was highly significant (*p* < 0.0001) with a non-significant lack-of-fit (*p* = 0.0689 > 0.05). The correlation coefficient (R^2^ = 0.9878) and adjusted R^2^ (0.9720) indicated good fit and predictive ability. ANOVA revealed that both GG/ι-C ratio (A) and glycerol content (C) significantly influenced σt (*p* < 0.01), with F-values of 132.92 and 140.47, respectively (Table S1). For elongation at break (εt), the fitted model was

εt = 6.17 − 0.88A + 0.010B + 1.10C − 0.14AB + 0.49AC + 0.36BC − 0.78A^2^ − 1.10B^2^ − 0.79C^2^.

The model was highly significant (*p* < 0.0001) with a non-significant lack-of-fit (*p* = 0.2901 > 0.05) [37]. The R^2^ (0.9825) and adjusted R^2^ (0.9601) confirmed good fit and predictive capability. ANOVA indicated that GG/ι-C ratio (A) and glycerol content (C) significantly affected εt (*p* < 0.01), with F-values of 86.68 and 133.98, respectively (Table S2). For light transmittance (T), the fitted model was

T = 80.11 − 1.04A + 0.17B − 1.89C − 0.10AB + 0.92AC − 0.88BC − 1.57A^2^ − 2.93B^2^ − 3.33C^2^.

The model was highly significant (*p* < 0.0001) with a non-significant lack-of-fit (*p* = 0.2387 > 0.05). The R^2^ (0.9849) and adjusted R^2^ (0.9654) demonstrated good fit and predictive ability. ANOVA revealed that glycerol content (C) significantly influenced T (*p* < 0.01), with an F-value of 88.15 (Table S3). Overall, the response-surface results showed that the GG/ι-C ratio, potassium citrate/sodium carbonate ratio, and glycerol content jointly affected the film properties. The significant quadratic terms indicated non-linear factor–response relationships and the existence of optimal intermediate ranges. The GG/ι-C ratio mainly influenced network compactness and film integrity, glycerol content affected chain mobility and intermolecular interactions, and the potassium citrate/sodium carbonate ratio may have regulated ion-mediated gelation and cross-linking within the polysaccharide network. In addition, the significant interaction between the GG/ι-C ratio and glycerol content suggested that the plasticizing effect of glycerol depended to some extent on the polysaccharide ratio.

A multi-objective optimization model was established using Design-Expert software. The optimal formulation parameters for HPMC-based capsules were obtained by numerical optimization as follows: 15.00% (*w*/*v*) HPMC, 0.26% (*w*/*v*) GG, 0.54% (*w*/*v*) ι-C, 0.051% (*w*/*v*) potassium citrate, 0.045% (*w*/*v*) sodium carbonate, and 0.49% (*w*/*v*) glycerol (plasticizer). The tensile strength, elongation at break, and light transmittance predicted by the fitted model were 30.28 MPa, 6.37%, and 80.35%, respectively. Repeat experiments were carried out to verify the reliability of the optimized formulation. The measured values were 29.67 ± 0.43 MPa for tensile strength, 6.72 ± 0.31% for elongation at break, and 79.83 ± 0.24% for light transmittance. The experimental values were close to the predicted values, indicating that the optimized formulation parameters obtained by response surface methodology were reliable and feasible.

### 2.4. Process Optimization of HPMC-Based Capsules

#### 2.4.1. Determination of Dipping Temperature

**Figure 3:**
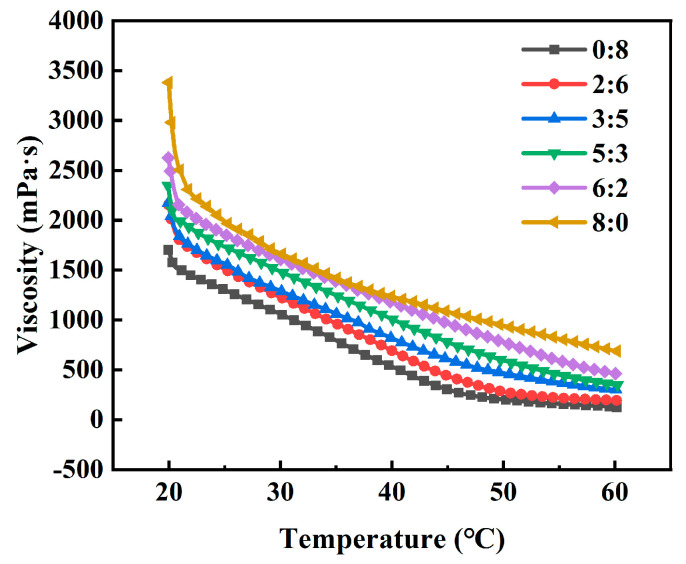
Temperature-modulated viscosity behavior of blended systems.

After completing the screening of formulation components, the dipping temperature of the gum solution became a critical parameter affecting the stability of the dipping process and the molding quality of the capsules. Figure 3 shows the viscosity-temperature curves of the blended gum solutions with different GG/ι-C ratios. The viscosity of all blended systems gradually decreased with increasing temperature, which could be attributed to the conformational transition of polysaccharides. At low temperatures, both GG and ι-C adopted helical conformations, where intermolecular hydrogen bonds and ionic bridging between helices enhanced molecular interactions, increasing flow resistance and leading to higher viscosity. As the temperature increased, GG and ι-C gradually underwent a helix-to-coil transition, forming random coil conformations [38,39]. The intermolecular spacing expanded, reducing the flow resistance between polysaccharide chains, which macroscopically manifested as a decrease in the viscosity of the gum solution. Notably, at the same temperature, the viscosity of the gum solution increased with increasing GG content, indicating that GG contributed more to the gel network density than ι-C under the test conditions. During the dipping process, two competing requirements must be balanced: avoiding excessive viscosity caused by premature gelation, while ensuring sufficient mixing and cross-linking between components. Therefore, the range of 55–60 °C was determined as the optimal dipping temperature for the GG/ι-C blended system. At this temperature, the viscosity of the gum solution was moderate, which not only met the fluidity requirements of the dipping process but also maintained sufficient gel strength to ensure stable capsule molding.

#### 2.4.2. Optimization of Drying Process Parameters

**Figure 4:**
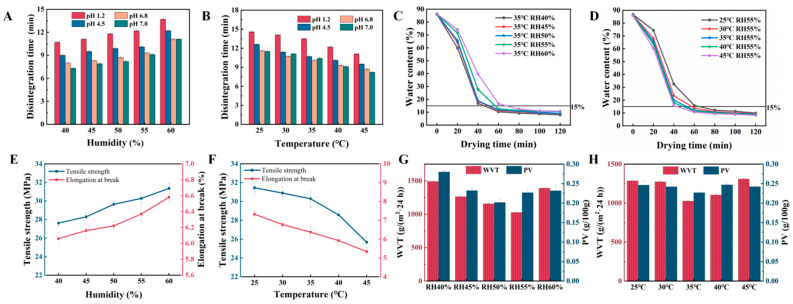
Effect of drying on capsule performance: Effect of drying humidity (**A**) and temperature (**B**) on the disintegration time of capsules. Effect of drying humidity (**C**) and temperature (**D**) for moisture content analysis during drying. Effect of drying humidity (**E**) and temperature (**F**) for effects on capsule film mechanical properties. Effect of drying humidity (**G**) and temperature (**H**) on water vapour transmission (WVT) and oxygen permeability (PV) of capsule films.

The drying process is a critical step in the production of HPMC-based hard capsules, as it directly influences their physical properties, stability, and bioavailability. Common drying methods include vacuum freeze-drying, fluidized bed drying, and hot air drying [40]. Among these, vacuum freeze-drying is associated with high cost and long processing time, while fluidized bed drying imposes certain requirements on the particle size of the material being dried [40]. Therefore, hot air drying was selected as the method for drying the HPMC-based hard capsules in this study. The disintegration time of capsules in four pH media (1.2, 4.5, 6.8, and 7.0) under different drying conditions is presented in Figure 4A,B. At a fixed drying temperature of 35 °C, increasing relative humidity progressively prolonged disintegration time (Figure 4A). This phenomenon may be attributed to reduced water evaporation rates under higher humidity, allowing more sufficient rearrangement of GG and ι-C chains within the capsule shell. This slower drying process potentially promotes tighter molecular chain alignment, forming a denser film structure with reduced porosity [41], thereby delaying water penetration and extending disintegration time. When relative humidity was fixed at 55%, increasing drying temperature progressively reduced disintegration time (Figure 4B). Higher temperatures may induce partial thermal pore formation in the polysaccharide-based capsule shell, creating more microporous structures that accelerate water penetration and subsequent disintegration [42]. To prevent capsule embrittlement and irregular fragmentation during disintegration, relative humidity should be maintained within 50–60% and drying temperature within 30–40 °C. These conditions effectively coordinate the pore structure of capsule shells, significantly enhancing disintegration rate while ensuring consistent disintegration performance across all four pH media.

Figure 4C illustrates the dynamic influence of drying time on the residual moisture content of capsule shells under various process parameters. At a fixed drying temperature of 35 °C, higher humidity resulted in slower drying rates and increased difficulty of water evaporation during drying from the capsule surface (Figure 4C). Elevated humidity promoted more uniform drying, reducing localized over-drying or uneven drying. During the drying-process analysis, the time required to reduce the residual moisture content below 15% was 42, 50, 52, 58, and 67 min at relative humidities of 40%, 45%, 50%, 55%, and 60%, respectively. At a fixed relative humidity of 55%, increasing drying temperature accelerated molecular chain movement and facilitated water evaporation during drying from the capsule shell [43] (Figure 4D). The time required to reduce the residual moisture content below 15% was 77, 60, 58, 49, and 40 min at drying temperatures of 25, 30, 35, 40, and 45 °C, respectively. Energy efficiency analysis revealed that drying at 25 °C reached the target moisture content, but the specific energy consumption remained high. Although drying at 45 °C significantly accelerated water removal, process stability decreased. Based on comprehensive evaluation, 30–40 °C and 50–60% relative humidity was identified as the optimal drying range, under which the residual moisture content of the capsule shells was maintained at approximately 7.9–9.0%.

The influence of drying conditions on mechanical properties of capsule films is shown in Figure 4E. At a fixed drying temperature of 35 °C, the tensile strength and elongation at break of the capsule films increased with the increase of relative humidity in the range of 40–60%. This phenomenon was attributed to the fact that higher relative humidity slowed down the drying rate, which reduced internal defects (e.g., microcracks) and improved the film-forming quality [44]. As illustrated Figure 4F, at a fixed relative humidity of 55%, the mechanical properties of the films decreased with the increase of drying temperature in the range of 25–45 °C. This was because higher temperatures intensified the movement of polysaccharide molecular chains, weakened the intermolecular interactions, and thus led to the embrittlement of the film material [45]. These results further verified the rationality of the drying parameters (30–40 °C and 50–60% relative humidity).

The effect of drying conditions on barrier properties of capsule films is presented in Figure 4G. With increasing relative humidity, both WVT and PV initially decreased and then increased (Figure 4). At low humidity, the substantial water vapor partial pressure difference between the capsule film and the environment facilitates rapid water diffusion outward, preventing the gel network from forming a compact structure and resulting in weak barrier properties. At high humidity, slow drying maintains the film structure in a prolonged hydrated state, similarly compromising barrier performance. Optimal barrier properties were observed at 55% relative humidity (WVT: 1026.75 ± 7.18 g/(m^2^·24 h)) and 50% relative humidity (PV: 0.202 ± 0.009 g/100 g). With increasing drying temperature, WVT and PV similarly exhibited an initial decrease followed by an increase (Figure 4H). At low temperatures, slow drying maintains the film structure in a prolonged hydrated state, preventing compact structure formation and resulting in elevated WVT and PV values. At high temperatures, intensified molecular chain mobility produces looser structures with compromised barrier properties, similarly elevating WVT and PV. Optimal barrier performance was achieved at 35 °C (WVT: 1026.75 ± 7.18 g/(m^2^·24 h), PV: 0.227 ± 0.001 g/100 g). It should be noted that PV was used here only as an indirect indicator of oxygen barrier performance, rather than a direct measurement of oxygen transmission rate.

**Figure 5:**
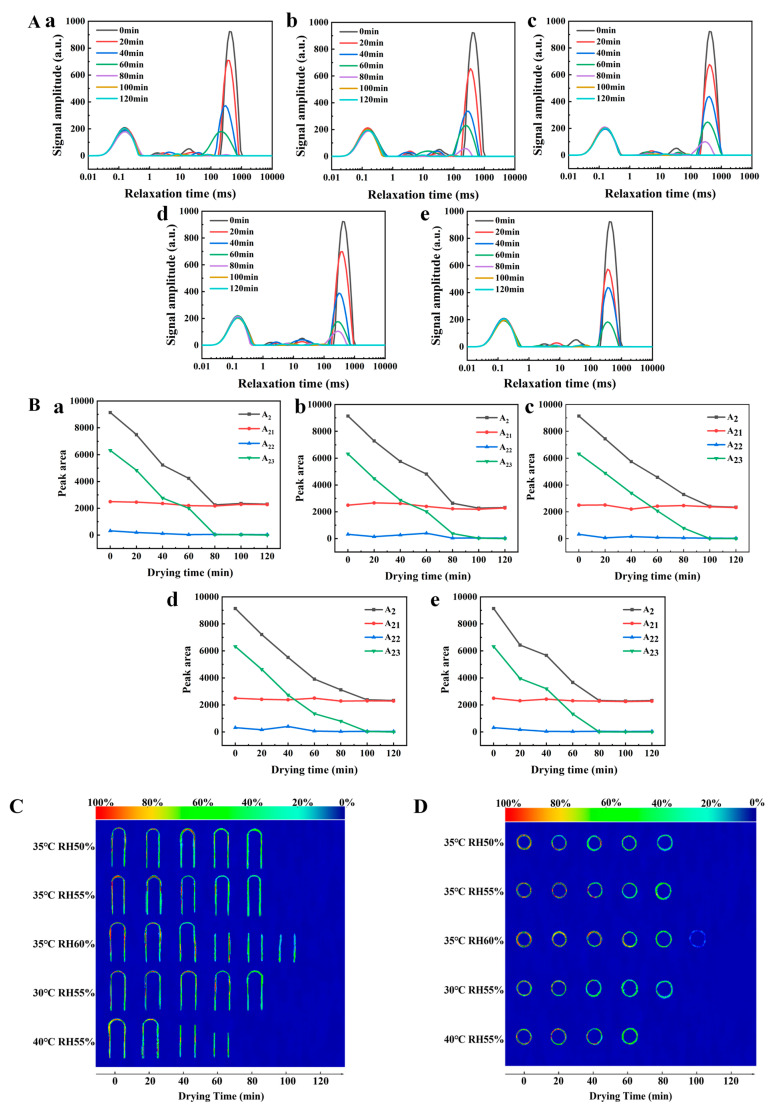
LF-NMR analysis and proton imaging of HPMC-based hard capsules under various drying regimes. (**A**) Distribution of transverse relaxation time (T2) indicating water mobility; (**B**) relative integration of relaxation peak areas. Sub-figures (**a**–**e**) in both (**A**) and (**B**) correspond to the following drying conditions: (**a**) 35 °C RH 50%, (**b**) 35 °C RH 55%, (**c**) 35 °C RH 60%, (**d**) 30 °C RH 55%, (**e**) 40 °C RH 55%; NMR images of capsules are displayed in (**C**) longitudinal section, (**D**) cross-section.

LF-MRI technology, featuring non-destructiveness and rapidity, could be used to real-time monitor moisture migration during the drying process. From the transverse relaxation time T_2_ curves in Figure 5A, it could be observed that the relaxation spectrum exhibited 3–4 characteristic peaks, which corresponded to bound water (T_21_, 0.01–10 ms), immobile water (T_22_, 10–100 ms), and free water (T_23_ > 100 ms) respectively, and the peak area could reflect the content of moisture in the corresponding phase [46]. In the undried gel samples, free water with a relaxation time of approximately 1000 ms accounted for 70% of the total moisture content, while immobile water with a relaxation time of approximately 16 ms accounted for 30%, indicating that the gel matrix exerted differential binding effects on water molecules.

Combining Figure 5A,B (transverse relaxation peak area diagram), it was found that with the continuation of the drying process, the total peak area A_2_ (total moisture content) decreased continuously, and each relaxation component migrated to the short time domain. This confirmed that during the drying process, water not only evaporated outward, but also underwent phase transition within the capsule film. In the early stage of drying, the free water peak area A_23_ decreased rapidly, while the immobile water peak area A_22_ increased slightly. This was mainly because part of the free water with relatively weak binding force was removed first, and another part of the free water was bound by polysaccharide molecular chains and converted into immobile water [47]. In the middle stage of drying (40–60 min), A_22_ first increased and then decreased, since immobile water became the main object of moisture removal, and part of it was converted into bound water. In the late stage of drying, only bound water remained in the system. At a fixed relative humidity of 55%, the higher the drying temperature, the faster the water evaporated from the capsule film during drying, which was consistent with the previous moisture content test results.

Figure 5C (longitudinal cross-section) and Figure 5D (horizontal cross-section) were proton density pseudo-color images of the capsules during the drying process. The color changed from red to green, representing a gradual decrease in proton density. These images intuitively reflect the moisture distribution. With the extension of drying time, the red area (high proton density, corresponding to free water) gradually narrowed, and the green area (low proton density, corresponding to bound water) increased continuously. Moreover, the red area shrank from the bottom edge of the capsule to the two walls, indicating that water evaporated first from the bottom and then from the two side walls. The experimental results showed that at a fixed relative humidity of 55%, no proton signal was detected in the samples after drying at 30 °C for 80 min, 35 °C for 80 min, and 40 °C for 60 min; at a fixed drying temperature of 35 °C, no proton signal was detected in the samples after drying at RH 50% and RH 55% for 80 min, and RH 60% for 100 min. These results confirmed that the higher the temperature, the faster water evaporated during drying, and the higher the humidity, the more uniform the drying. Based on the comprehensive experimental results of disintegration time, the residual moisture content, mechanical properties, and barrier properties, combined with the microscopic analysis of LF-MRI technology, the optimal drying process parameters were finally determined to be 35 °C and 55% relative humidity. These conditions were selected to achieve uniform drying, improve the comprehensive performance of products, reduce production energy consumption, and ensure process stability. This process could enable the capsules to disintegrate rapidly within 15 min in four pH media, control the residual moisture content below 15%, and possess good mechanical properties and barrier properties, which fully met the requirements of industrial production.

### 2.5. Physicochemical Properties and Storage Stability

HPMC-based capsules were prepared using the optimized formulation and process parameters (Table 3). To evaluate storage stability, the capsules were stored under ambient conditions at 50% relative humidity. Physicochemical properties were assessed at 0, 30, and 180 days of storage, and the results are summarized in Table 4. Throughout the 180-day storage period, all tested parameters remained well within the specifications of the Chinese Pharmacopoeia (2025) for hypromellose hard capsules [23]. Notably, the disintegration time in acidic medium (pH 1.2) decreased from ≤13 min at day 0 to ≤12 min at day 180, while remaining substantially below the pharmacopoeial limit of 15 min. The capsules exhibited excellent mechanical integrity, with no fractures observed in friability testing (0/50 capsules) and no separation or leakage in tightness testing (0/10 capsules) throughout the storage period. Loss on drying ranged from 6.01% to 6.85%, well below the ≤8% limit, and residue on ignition remained under 3% at all time points. Heavy metal content consistently complied with the ≤20 ppm requirement. These results demonstrate that the HPMC-based capsules produced using the optimized formulation and process exhibit excellent storage stability, maintaining consistent physicochemical performance under standard storage conditions for at least 180 days. This confirms the reliability of the developed capsules for practical production and application, but their disintegration rate still has a certain gap with the cutting-edge fast-disintegrating capsules. Song et al. realized disintegration within 5 min through Pullulan-Hyaluronan composite films, which offers an important reference for the further optimization of our formulation to achieve faster disintegration [4].

### 2.6. In Vitro Drug Release Study

According to the Biopharmaceutics Classification System (BCS), drugs are categorized into four classes based on their aqueous solubility and intestinal permeability characteristics [48]. Due to their poor aqueous solubility, Class II and IV drugs were excluded from this study to focus on the release profiles of highly soluble compounds [49]. Therefore, a Class I drug (cefradine) and a Class III drug (ranitidine hydrochloride) were selected as model drugs to evaluate the dissolution performance of the developed capsules under the tested conditions.

**Figure 6:**
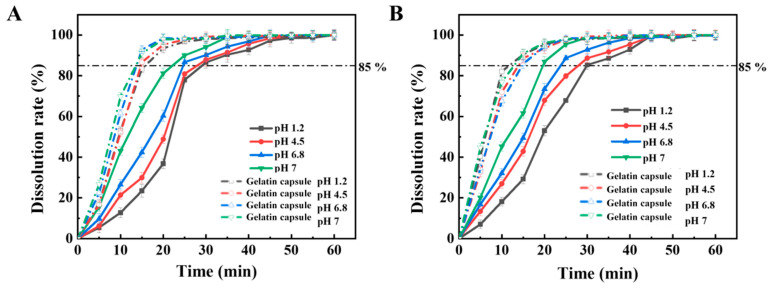
Dissolution profiles of cefradine (**A**) and ranitidine hydrochloride (**B**) in different dissolution media (artificial gastric fluid, pH = 1.2), acetate–sodium acetate buffer (pH = 4.5), phosphate buffer (pH = 6.8) and distilled water (pH = 7.0).

The in vitro drug release profiles of HPMC-based capsules compared with conventional gelatin capsules are presented in Figure 6. For cefradine-loaded HPMC-based capsules, capsule rupture occurred within approximately 3–5 min (Figure 6A), indicating rapid disintegration and prompt onset of drug release. For ranitidine hydrochloride, a relatively limited release was observed during the first 5 min, with the majority of drug release occurring predominantly between 5–10 min (Figure 6B). This delayed release pattern for ranitidine hydrochloride may be attributed to its higher solubility and potential interactions with the polysaccharide matrix during the initial hydration phase. Both formulations fully complied with the dissolution requirements of the Chinese Pharmacopoeia (2025) [23]. Notably, in pH 1.2 and 4.5 media, the HPMC-based capsules exhibited relatively slower drug release compared to gelatin capsules. This phenomenon can be attributed to ionic interactions between the anionic sulfate groups of ι-C and the acidic buffer components, which may temporarily stabilize the gel network and retard drug diffusion [25]. Across all four tested media (pH 1.2, 4.5, 6.8, and 7.0), drug release from the HPMC-based capsules initiated at approximately 3–10 min, with cumulative release reaching ≥85% within 30 min. The tested HPMC-based capsules achieved nearly complete drug release by the end of the testing period, showing comparable in vitro drug release behavior to gelatin capsules for the two model drugs under the tested conditions. To quantitatively assess the similarity between dissolution profiles, the similarity factor (f_2_) method was employed. As summarized in Table 5, the f_2_ values for both cefradine and ranitidine hydrochloride exceeded 50 across all four pH media, confirming similarity between the dissolution profiles of the HPMC-based capsules and gelatin capsules. According to regulatory guidelines, f2 values within the range of 50–100 indicate dissolution similarity between the compared profiles [50]. These findings collectively demonstrate that the developed HPMC-based capsules exhibit rapid and pH-dependent disintegration behavior and, for the two tested highly soluble model drugs, show similar dissolution profiles to conventional gelatin capsules under the tested conditions. The capsules therefore represent a promising HPMC-based capsule system for pharmaceutical applications requiring rapid drug release under the tested conditions, although further evaluation with a broader range of drug classes is still needed.

### 2.7. SEM Analysis of Capsule Shell Disintegration

**Figure 7:**
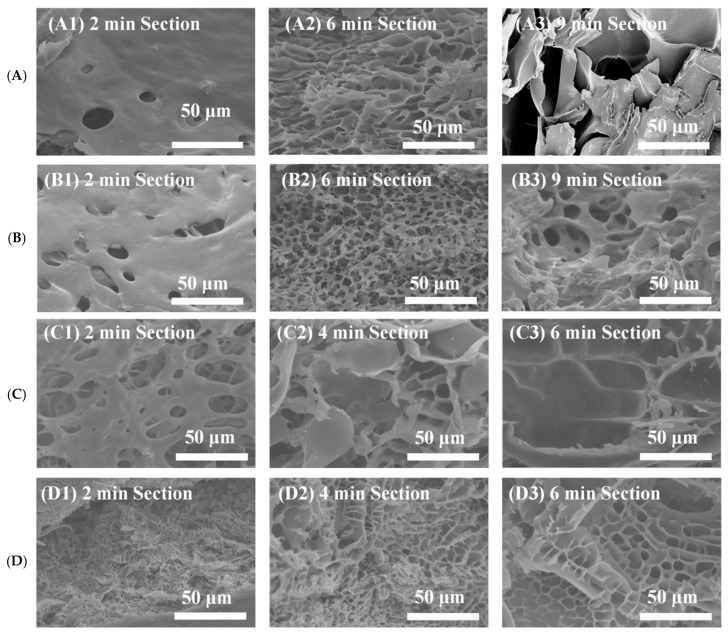
Cross-sectional scanning electron microscopy (SEM) images of HPMC/GG/ι-C capsule shells after disintegration in simulated gastrointestinal fluids with different pH values. (**A**) pH 1.2; (**B**) pH 4.5; (**C**) pH 6.8; (**D**) pH 7.0. The images were captured at 2 min (**A1**,**B1**,**C1**,**D1**), 4 min (**C2**,**D2**), 6 min (**A2**,**B2**,**C3**,**D3**), and 9 min (**A3**,**B3**), respectively.

SEM was employed to characterize the cross-sectional microstructural evolution of the HPMC-based hard capsules in simulated gastrointestinal fluids, thereby elucidating the microstructural basis underlying their rapid disintegration. At the initial stage (approximately 2 min), the capsule shells exhibited pronounced hydration-induced swelling. This behavior was primarily driven by the hydrophilic synergy of the GG/ι-C composite matrix, where polar moieties such as the carboxyl groups of GG and sulfate ester groups of ι-C facilitated rapid water binding and triggered gel network expansion [13]. At pH 1.2 (Figure 7(A1)), the cross-section remained relatively dense at 2 min, signifying an initial resistance to water penetration. This transient delay may be attributed to the protonation of GG carboxyl groups under strongly acidic conditions, a process that reduces chain hydrophilicity and induces localized network contraction [51]. As illustrated in Figure 7(A2,B2,C2,D2), the intermediate stage of disintegration (approximately 4–6 min) was characterized by a rapid proliferation of micropores. SEM analysis revealed a marked increase in both the density and dimensions of these pores, accompanied by an evident coalescence of adjacent cavities, which collectively signaled the onset of matrix dissolution. During this phase, the continuous infiltration of water molecules disrupted the hydrogen bonding and ionic cross-linking interactions between the GG and ι-C chains. This disruption led to the progressive relaxation and eventual breakdown of the originally compact three-dimensional network. In contrast, the samples immersed in pH 7.0 media (Figure 7D) exhibited the most rapid structural disintegration. A refined, sponge-like porous architecture emerged as early as 2 min, followed by an extreme thinning of the framework that approached the disintegration threshold at 4 min, ultimately culminating in complete structural fragmentation by 6 min. This accelerated process was attributed to the simultaneous deprotonation of the functional groups. The complete ionization of these moieties significantly increased the negative charge density within the polymer network, which intensified the interchain electrostatic repulsion. This heightened repulsion, coupled with the enhanced hydrophilicity of the matrix, facilitated rapid water infiltration and osmotic swelling, ultimately leading to the efficient disintegration of the gel framework [52].

Overall, the observed structural degradation across all pH media within 10 min was consistent with the macroscopic disintegration results, providing microstructural evidence for the rapid disintegration behavior of the developed capsule shells.

## 3. Materials and Methods

### 3.1. Materials

Gellan gum (GG, low acyl form, with molecular weight of 500 kDa and purity > 99%) was purchased from Fufeng Biotechnology Co., Ltd. (Urumqi, China). ι-Carrageenan (ι-C, food grade) was supplied by Lvxin (Putian, Fujian) Food Co., Ltd. (China), with a sulfate content of 25.9%, a viscosity of 0.056 Pa·s, and an Mw of 4.37 × 105 g/mol, with viscosity measurements taken at a 1.5% (*w*/*v*) concentration and 75 °C. Hydroxypropyl methylcellulose (HPMC, ZW-HPMC-2910E4) was obtained from Huzhou Zhanwang Pharmaceutical Co., Ltd. (Huzhou, China), with a methoxy content of 29.0%, a hydroxypropoxy content of 8.2%, a viscosity of 4.1 mPa·s, and Mw of 2.65 × 104 g/mol. The physicochemical parameters of GG, ι-C, and HPMC were based on supplier specifications and quality documents. Potassium citrate (≥98%) and sodium citrate (≥98%) were purchased from Shanghai Aladdin Biochemical Technology Co., Ltd. (Shanghai, China). Sodium carbonate and potassium carbonate (analytical reagent, AR) were supplied by Sinopharm Chemical Reagent Co., Ltd. (Shanghai, China). Sodium chloride, potassium chloride, glycerol (AR) and calcium chloride (AR) were obtained from Xilong Scientific Co., Ltd. (Shantou, China). Xylitol (food grade) was supplied by Shandong Futian Pharmaceutical Co., Ltd. (Shanghai, China). Sorbitol (≥98%) was purchased from Shanghai Aladdin Biochemical Technology Co., Ltd. (Shanghai, China). Polyethylene glycol (PEG)-200 and PEG-6000 (AR) were obtained from Xilong Scientific Co., Ltd. (Shantou, China), which were used as additives to regulate the viscosity of the gum solution. All chemicals and raw materials were used as received without further purification, and purified water used in all experiments was prepared in-house.

### 3.2. Preparation of Gum Solution

Deionized water (400 mL) was charged into a 1 L beaker and placed in a constant-temperature water bath equipped with a magnetic stirrer. While maintaining the temperature at 40 ± 2 °C, sodium carbonate (0.20 g), potassium citrate (0.20 g), and GG (0.00, 0.80, 1.20, 2.00, 2.40, and 3.20 g) were sequentially incorporated under continuous stirring for preliminary homogenization. The system was then gradually heated to 80 ± 2 °C, followed by the stepwise addition of HPMC (60 g) and ι-C (3.20, 2.40, 2.00, 1.20, 0.80, and 0.00 g, respectively). The mixture was stirred at constant temperature until the gum solution was fully dispersed and dissolved. The water evaporated during the heating process was recorded and supplemented with deionized water. The beaker was sealed with plastic wrap, and after 3 h, the temperature was cooled to 60 °C for gum preservation, resulting in gum solutions with different GG/ι-C ratios (0:8, 2:6, 3:5, 5:3, 6:2, 8:0).

### 3.3. Preparation of Capsule Films

Gum solutions were prepared according to the method described in Section 3.2. After preparation, 13 mL of the gum solution was taken and evenly spread on a preheated plastic petri dish (13 × 13 cm). Subsequently, the petri dish with the spread gum solution was placed in an oven with a constant temperature of 35 °C for continuous drying for 2.5 h. The formed film was carefully peeled off and hermetically stored for subsequent determination.

### 3.4. Preparation of Capsules

Gum solutions were prepared according to the method described in Section 3.2, which were used to fabricate GG/ι-C composite gel HPMC-based hard capsules. First, the temperature of the prepared gum solution was cooled to the dipping temperature. Subsequently, No. 0 capsule molds were taken, and mold release oil was evenly coated on their surfaces. The molds were then vertically immersed into the gum solution and kept for 1–2 s to dip the gum solution. After that, the molds were taken out and quickly flipped up and down several times to ensure uniform distribution of the gum solution on the molds, thereby forming a transparent and homogeneous gel. After the dipping operation, the molds were placed in an oven at 35 °C for drying for 2.5 h. Upon completion of drying, processes such as shell stripping, cutting, and fitting were performed sequentially to finally prepare the capsules.

### 3.5. Measurement of Capsule Shell Thickness and Selection of Dipping Temperature

The prepared gum solution was cooled to an appropriate temperature, followed by dipping, drying, shell stripping, cutting, and fitting. The shell thickness of size 0 capsules was specified as 0.085–0.115 mm [53]. The thickness of capsule shells was determined using a digital thickness gauge (Model 543-400BS, Mitutoyo Corp., Kawasaki, Japan) with a resolution of 0.001 mm and a measurement accuracy of ±0.002 mm. Whether the thickness met the specified standard was evaluated, and the dipping temperatures corresponding to qualified capsule shells were recorded.

### 3.6. Determination of Mechanical Properties

The mechanical properties of the capsule films were determined according to a slightly modified method reported by Dong et al. [13]. The dried capsule films were cut into rectangular specimens with a width of 10 mm and a length of 80 mm. The thickness of each specimen was measured using a digital thickness gauge (Model 543-400BS, Mitutoyo Corp., Kawasaki, Japan) with a resolution of 0.001 mm and an accuracy of ±0.002 mm. Mechanical tests were carried out using a universal tensile testing machine (Model CMT4204, Sansi (Shanghai) Enterprise Development Co., Ltd., Shanghai, China). The initial grip distance (*L*_0_) was set at 50 mm, and the strain rate was controlled at 5 mm/min. Tensile strength (σ_t_) and elongation at break (*ε_t_*) were recorded automatically during the test. All measurements were performed in triplicate to minimize random errors, and the mean values were used as the final results. Tensile strength (σ_t_) and elongation at break (*ε_t_*) were calculated according to Equations (1) and (2), respectively,

(1) $$\sigma_{t}=\frac{F}{S}$$

(2) $$\varepsilon_{t}=\frac{L-L_{0}}{L_{0}}\times 100\%$$

where *F* is the maximum tensile force that the capsule film can withstand at break (N); *S* is the original cross-sectional area of the capsule film (m^2^); *L_0_* is the initial length of the capsule film (mm); *L* is the length of the film at break (mm).

### 3.7. Determination of Disintegration Time

Six HPMC-based hard capsules were taken and filled with talcum powder. The disintegration time was determined in accordance with the Disintegration Test Method (General Rule 0921) of the Chinese Pharmacopoeia (2025) [23]. The test was carried out using an intelligent disintegration tester (Model ZB-1E, Tianda Tianfa Technology Co., Ltd., Tianjin, China) with a baffle. The time required for each capsule to completely disintegrate was observed and recorded.

### 3.8. Determination of Light Transmittance

The light transmittance of capsule films was determined using an ultraviolet–visible (UV-Vis) spectrophotometer (Model UV-8000, Shanghai Yuanxi Instrument Co., Ltd., Shanghai, China). The specific operation steps were as follows: the dried film samples were cut into rectangular specimens with a size of 1 × 4 cm, ensuring a flat and wrinkle-free surface; the detection wavelength was set at 600 nm, air was used as the reference, and baseline correction was performed before the test; the specimens were placed in the sample cell, and the light transmittance was measured at an ambient temperature of 25 ± 2 °C [54]. All measurements were performed in triplicate for each sample, and the mean values were taken as the final results.

### 3.9. Formulation Optimization of the HPMC-Based Hard Capsules

#### 3.9.1. Single-Factor Experiment Design

Single-factor experiments were conducted to investigate the effects of different component ratios and additive concentrations on the mechanical properties and light transmittance of capsule films. The experimental variables were set as follows: the total mass fraction of GG and ι-C was fixed at 0.80% (*w*/*v*), and their mass ratios were set to 1:7, 2:6, 3:5, 4:4, 5:3, 6:2, 7:1, and 8:0, respectively; the total mass fraction of potassium citrate and sodium carbonate was fixed at 0.096% (*w*/*v*), with their mass ratios set to 0:8, 1:7, 2:6, 3:5, 4:4, 5:3, 6:2, 7:1, and 8:0, respectively; the mass fractions of glycerol (plasticizer) were set to 0.00%, 0.25%, 0.50%, 0.75%, 1.00%, and 1.25% (*w*/*v*), respectively. Other experimental conditions were kept constant during the single-factor experiments to ensure the reliability of the results.

#### 3.9.2. Response Surface Experiment Design

A Box–Behnken design combined with response surface methodology (RSM) was employed to systematically optimize the formulation of HPMC-based hard capsules. The mass ratio of GG/ι-C, the mass ratio of potassium citrate/sodium carbonate, and the mass fraction of glycerol (plasticizer) were selected as independent variables, and their key parameter ranges were determined based on the results of single-factor preliminary experiments. σ_t_/MPa, *ε_t_*/%, and light transmittance at 600 nm (T/%) were chosen as response indicators. The optimal formulation parameters were obtained by numerical optimization method to achieve the comprehensive improvement of the performance of HPMC-based hard capsules.

### 3.10. Effect of Temperature on Viscosity of Composite Gum Solution

The viscosity-temperature behavior of the gum solution was determined according to the method of Tu et al. with slight modifications [55]. The temperature control platform of an intelligent rheometer (Model MCR-302, Anton Paar Trading Co., Ltd., Shanghai, China) was preset to 20 ± 0.5 °C, and the test mode was set to constant strain (1.0%) and constant shear rate (100 s^−1^). A 1 mL plastic pipette was used to transfer the gum solution to be tested, which was prepared according to the method described in Section 3.2. The gum solution was evenly spread on the surface of the sample stage with a constant temperature. The heating program was started, and the temperature of the system was gradually increased from the initial 20 °C to 60 °C at a linear rate of 5 °C/min, with the preset shear conditions maintained throughout the process. The viscosity change of the system was monitored in real time by the built-in sensor of the rheometer, and the viscosity-temperature relationship curve was recorded synchronously.

### 3.11. Determination of Moisture Content Under Different Drying Conditions

Ten capsule molds (No. 1 to No. 10) were prepared, and their weights were measured before and after gum dipping. The molds were then placed in a DYG-9036A constant temperature and humidity oven (Shanghai Jinghong Co., Ltd., Shanghai, China) for drying, with the drying temperature and relative humidity (RH) of the oven adjusted as experimental variables. The molds were weighed at 20, 40, 60, 80, 100, and 120 min during the drying process. Each of the ten molds was weighed and calculated separately, and the mean value was taken as the final result. The moisture content of the capsules was calculated according to Equation (3):

Moisture content (%) = (m_1_ − m_t_)/(m_1_ − m_0_) × 100 (3)

where m_0_ is the mass of the mold before gum dipping (g); m_1_ is the mass of the mold after gum dipping (g); m_t_ is the mass of the mold after drying for t minutes (g).

### 3.12. Barrier Property Test Under Different Drying Conditions

#### 3.12.1. Determination of Water Vapor Transmission (WVT)

The WVT of film samples was determined by the cup-weight gain method, slightly modified from the method reported by Costa et al. [56]. Anhydrous calcium chloride (15.00 ± 1.00 g) with a particle size of 0.60–2.36 mm was placed into a moisture permeability cup. Prior to use, the anhydrous calcium chloride was dried in an oven at 200 ± 2 °C for 2 h. A smooth and flat part of the capsule film was selected and placed at the opening of the cup body. A mixture of 85% paraffin and 15% beeswax (mass ratio) was heated and melted for edge sealing. The initial weight of the moisture permeability cup was recorded with an accuracy of 0.1 mg. The assembled moisture permeability cup was placed in a desiccator at a temperature of 23 ± 0.5 °C and a relative humidity of 90 ± 2%. The cup was taken out every 12 h and weighed using an electronic balance, with the data recorded to an accuracy of 0.1 mg. The WVT value of the sample was calculated based on the weight increment of the moisture permeability cup. All measurements were performed in triplicate for each sample, and the mean value was taken as the final result. The specific mathematical expression is shown in Equation (4):

WVT = (24 × Δm)/(A × t) (4)

where WVT is the water vapor transmission (g/(m^2^·24 h)); Δm is the mass change of the moisture permeability cup within time t (g); A is the water vapor transmission area of the specimen (m^2^); t is the time interval between two stable mass changes (h).

#### 3.12.2. Determination of Oxygen Permeability (PV)

Fresh first-grade soybean oil (10.00 g) was accurately weighed and placed into a 50 mL centrifuge tube. The mouth of the centrifuge tube was hermetically sealed with a capsule film sample to ensure good sealing performance. Subsequently, the sealed centrifuge tube was placed at room temperature for oxidation treatment over 14 days. After the oxidation treatment, the peroxide value of the soybean oil was determined by titration with a 0.01 mol/L standard sodium thiosulfate (Na_2_S_2_O_3_) solution. To ensure the accuracy of the experimental data, all measurements were performed in triplicate, and the mean value of the three parallel tests was taken as the final result [57]. PV was calculated according to Equation (5):

PV = [(V − V_0_) × c × 0.1269]/m × 100 (5)

where PV is the peroxide value (g/100 g); V is the volume of the standard Na_2_S_2_O_3_ solution consumed by the sample (mL); V_0_ is the volume of the standard Na_2_S_2_O_3_ solution consumed by the blank test (mL); c is the concentration of the standard Na_2_S_2_O_3_ titration solution (mol/L); m is the mass of the first-grade soybean oil used for titration (g); 0.1269 is the mass of iodine equivalent to 1.00 mL of the standard Na_2_S_2_O_3_ solution [1.0 mol/L].

### 3.13. Determination of Transverse Relaxation Time by Low-Field Nuclear Magnetic Resonance (LF-MRI)

Gum dipping was performed using 8–10 plastic capsule molds, which were then placed in a constant temperature and humidity oven for drying. The capsules were subjected to different drying temperatures (30, 35, and 40 °C) while maintaining a constant relative humidity (RH) of 55%. In addition, the capsules were exposed to different relative humidities (50, 55, and 60%) while keeping the temperature constant at 35 °C. Samples were randomly taken from the constant temperature and humidity oven at 20-min intervals, placed into NMR tubes, and the NMR tubes containing the samples were inserted into a LF-MRI analyzer (Model NMI20-060H-I, Suzhou Niumai Analysis Instrument Co., Ltd., Suzhou, China) for LF-MRI scanning. The scanning was continued until a constant weight was achieved or a weak signal was obtained. The key test parameters of the LF-MRI analyzer were set as follows: sequence (SEQ): CPMG; main frequency (SF): 21 MHz; frequency offset (O1): 59,548.46 Hz; 90° pulse width (P1): 5.00 μs; number of sampling points (TD): 187526; sampling frequency (SW): 250 KHz; radio frequency delay (RFD): 0.080 ms; analog gain (RG1): 20.0 dB; digital gain (DRG1): 3; preamplification factor (PRG): 1; waiting time (TW): 5000.00 ms; 180° pulse width (P2): 10.00 μs.

### 3.14. Low-Field Nuclear Magnetic Resonance Imaging (NMRI)

Proton density images of the vertical and cross-sections of the samples were acquired using the multi-slice spin-echo sequence in the low-field nuclear magnetic resonance imaging analysis software, slightly modified from the method reported by Ojha et al. [58]. First, an oil sample was placed into the instrument for calibration. The NMR tube containing the plastic capsule mold with gum dipping was placed at the center of the magnet box for signal acquisition. The key test parameters of the NMRI analyzer were set as follows: number of slices (Slices): 1; slice width: 2 mm; slice gap: 1 mm; number of averages (Average): 2; repetition time (TR): 2000.00 ms; echo time (TE): 18.125 ms; phase size: 192; read size: 256.

### 3.15. Determination of Physicochemical Properties of HPMC-Based Hard Capsules

The inspection of the composite HPMC-based hard capsules was carried out with reference to the quality standards for HPMC-based hard capsules specified in the Chinese Pharmacopoeia (2025) [23]. The physicochemical properties of the HPMC-based hard capsules were systematically detected and evaluated in accordance with the relevant requirements of the pharmacopoeia to ensure the quality and usability of the prepared capsules.

### 3.16. Drug Dissolution Experiment

#### 3.16.1. Drug Dissolution Experiment and Determination of Dissolution Rate

To evaluate the drug release characteristics of the capsules, cefradine and ranitidine hydrochloride were selected as model drugs and loaded into the prepared GG/ι-C composite HPMC-based hard capsules, with 0.25 g of drug powder filled in each capsule [59]. The dissolution rate of the capsules was determined using a dissolution tester (Model ZRS 8GD, Tianda Tianfa Technology Co., Ltd., Tianjin, China) in four types of pH dissolution media (pH = 1.2, 4.5, 6.8, 7.0). Each of the six dissolution cups was filled with 900 mL of dissolution medium and maintained at 37 ± 0.5 °C. A capsule was placed in each basket, and 2 mL of sample was withdrawn at predetermined intervals. The withdrawn sample was rapidly filtered through a 0.22 μm filter membrane into a 2 mL centrifuge tube, and the volume of the withdrawn sample was immediately replaced with fresh preheated dissolution medium (37 ± 0.5 °C) [60]. After appropriate dilution with the corresponding dissolution medium, the samples were analyzed using an ultraviolet-visible (UV-Vis) spectrophotometer. The absorbance of the dissolved cefradine samples was measured at a wavelength of 255 nm, while the absorbance of the dissolved ranitidine hydrochloride samples was measured at 314 nm [23]. The concentration of the drug in each sample was calculated using the standard curve, and the dissolution rate curve was plotted accordingly.

#### 3.16.2. Similarity of Dissolution Curves

The similarity of dissolution curves was compared using the similarity factor (f_2_) method, which is a model-independent approach [61]. The average dissolution amount of the test samples (the HPMC-based hard capsules) was compared with that of the reference samples (gelatin capsules). The specific mathematical expression is shown in Equation (6):

f_2_ = 50 × lg{[1 + (1/n)Σ_t=1_^n^(R_t_ − T_t_)^2^]^−0.5^ × 100} (6)

where R_t_ is the average dissolution amount of the reference sample at time t (%); T_t_ is the average dissolution amount of the test sample at time t (%); n is the number of sampling time points, with n = 6.

### 3.17. Scanning Electron Microscopy (SEM)

HPMC-based hard capsules were prepared using the optimized formulation. To investigate the microstructural evolution during disintegration, samples were collected at specific intervals from four media with different pH values (1.2, 4.5, 6.8, and 7.0). Specifically, sampling was performed at 2, 6, and 9 min for the media at pH 1.2 and 4.5, and at 2, 4, and 6 min for those at pH 6.8 and 7.0. Immediately upon collection, the samples were immersed in liquid nitrogen for cryogenic fracturing. Subsequently, the fractured specimens were dehydrated using a freeze-dryer. For SEM, specimens were sputter-coated with gold and imaged at 5.0 kV (SU5000, Hitachi High-Technologies, Tokyo, Japan).

### 3.18. Statistical Analysis

Each experiment was performed in triplicate to ensure reproducibility and reliability. Statistical differences among samples were determined using one-way ANOVA followed by Duncan’s multiple range test (*p* < 0.05), performed with SPSS 16.0.

## 4. Conclusions

In this study, high-performance HPMC-based hard capsules were developed and systematically optimized using an HPMC/GG/ι-C ternary matrix. The optimal formulation was determined as HPMC 15.00% (*w*/*v*), gellan gum (GG) 0.26% (*w*/*v*), ι-carrageenan (ι-C) 0.54% (*w*/*v*), potassium citrate 0.051% (*w*/*v*), sodium carbonate 0.045% (*w*/*v*), and glycerol 0.49% (*w*/*v*), and the optimal drying condition was confirmed to be 35 °C with 55% relative humidity. Through formulation screening and comprehensive drying process optimization, HPMC hard capsules that meet the requirements of the Chinese Pharmacopoeia (2025) and exhibit rapid disintegration were successfully prepared. The final product could disintegrate rapidly within 15 min in four pH media (pH 1.2, 4.5, 6.8, and 7.0) and exhibited excellent mechanical strength, good barrier properties, uniform molding and stable processability. Dissolution studies using the two highly soluble model drugs, cefradine and ranitidine hydrochloride, showed cumulative release above 85% within 30 min and dissolution similarity to conventional gelatin capsules under the tested conditions. SEM observations further suggested that the rapid disintegration of the capsule shells may be associated with hydration-induced swelling, micropore formation, and progressive loosening of the GG/ι-C network under simulated gastrointestinal pH conditions. These results demonstrate that the developed formulation is a promising HPMC-based capsule system with rapid disintegration across simulated gastrointestinal pH conditions. However, further studies are still needed to evaluate a broader range of drug classes, especially poorly soluble compounds, and to clarify the detailed molecular mechanism of disintegration.

## Figures and Tables

**Table 1:** Experimental factors and levels for response surface methodology design.

No.	Factor	Levels		
		−1	0	1
A	GG: ι-C	2:6	3:5	4:4
B	Potassium citrate: Sodium carbonate	3:5	4:4	5:3
C	Glycerol (%)	0.25	0.50	0.75

**Table 2:** Experimental program design and data.

No.	A	B	C	σt/MPa	εt/%	T/%
1	0	−1	1	27.18	5.22	72.23
2	1	0	1	27.57	5.09	73.23
3	0	0	0	29.24	6.25	79.56
4	−1	1	0	27.47	5.34	76.45
5	1	0	−1	17.09	2.38	74.69
6	−1	−1	0	26.74	5.13	76.82
7	1	−1	0	21.02	3.47	74.87
8	1	1	0	20.02	3.21	74.31
9	0	−1	−1	21.03	3.45	74.53
10	0	0	0	29.31	6.42	79.62
11	−1	0	1	26.86	5.63	73.43
12	0	0	0	29.85	5.81	80.25
13	−1	0	−1	26.57	4.97	78.58
14	0	1	−1	21.14	2.92	77.48
15	0	1	1	26.22	5.96	71.66
16	0	0	0	30.07	6.26	79.89
17	0	0	0	30.04	6.25	80.65

**Table 3:** Final optimized formulation and process conditions for HPMC-based hard capsules.

Parameter	Optimized Condition
HPMC	15.00% (*w*/*v*)
GG	0.26% (*w*/*v*)
ι-C	0.54% (*w*/*v*)
Potassium citrate	0.051% (*w*/*v*)
Sodium carbonate	0.045% (*w*/*v*)
Glycerol	0.49% (*w*/*v*)
Drying temperature	35 °C
Relative humidity	55%

**Table 4:** Measurement results of physical and chemical indexes of HPMC-based hard capsules.

Physicochemical Property		0 Day	30 Day	180 Day	Chinese Pharmacopoeia (2025) Requirement
Wall thickness/mm		0.102 ± 0.006	0.103 ± 0.009	0.103 ± 0.017	0.085–0.115
Disintegration time/min	pH = 1.2	≤13	≤13	≤12	<15
	pH = 4.5	≤13	≤12	≤12	<15
	pH = 6.8	≤11	≤11	≤10	<15
	pH = 7.0	≤10	≤10	≤10	<15
Friability/grain		0/50	0/50	0/50	≤2/50
Tightness/grain		0/10	0/10	0/10	<1/10
Loss on drying/%		6.33 ± 0.12	6.01 ± 0.26	6.85 ± 0.13	≤8
Residue on ignition/%		1.67 ± 0.16	1.64 ± 0.18	1.86 ± 0.21	<3
Heavy metal content/ppm		Conforms	Conforms	Conforms	≤20

**Table 5:** Dissolution curve similarity factors (f2).

Dissolution Medium	Cefradine	Ranitidine Hydrochloride
pH = 1.2	54.35	51.43
pH = 4.5	55.64	55.93
pH = 6.8	56.73	69.20
pH = 7.0	72.94	90.88

## Data Availability

The datasets used and/or analyzed during the current study are available from the corresponding authors on reasonable request.

## Supplementary Materials

The following supporting information can be downloaded at https://www.mdpi.com/article/10.3390/md24050162/s1. Figure S1: Samples of prepared HPMC-based hard capsules; Table S1: Analytical model parameters for tensile strength; Table S2: Analytical model parameters for breaking elongation; Table S3: Analytical model parameters for transmittance; Table S4: The standard curve regression equation and correlation coefficient of Cefradine; Table S5: The standard curve regression equation and correlation coefficient of Ranitidine [62,63].

## Abbreviations

The following abbreviations are used in this manuscript:

HPMC	Hydroxypropyl methylcellulose
GG	Gellan gum
ι-C	Iota-Carrageenan
WVP	Water vapor permeability
PV	Peroxide value
SEM	Scanning electron microscope
LF-MRI	Low-Field Nuclear Magnetic Resonance
