# Supplementary material for: Development of HPMC-Based Hard Capsules with Rapid Disintegration Across Simulated Gastrointestinal pH Conditions: Formulation Design, Process Optimization, and Disintegration Mechanism of the HPMC/GG/ι-C Ternary System
Source: Mar Drugs. 2026 May 2;24(5):162. doi: 10.3390/md24050162 (PMC13208135; doi:10.3390/md24050162)
Supplement: Supplementary file 1 [file marinedrugs-24-00162-s001.zip › marinedrugs-4217815-supplementary.pdf]

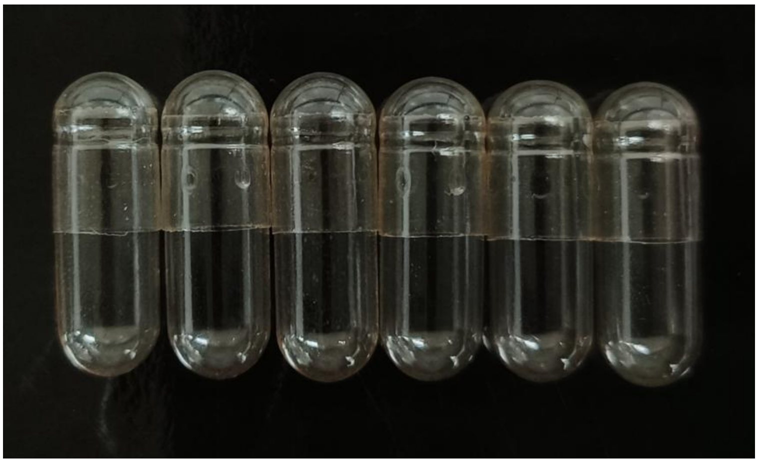

Figure S1. Samples of prepared HPMC hard capsules

Table S1. Analytical model parameters for tensile strength

Source	Sum of	df	Mean	F	P
Model	260.58	9	28.95	62.81	< 0.0001
A	61.27	1	61.27	132.92	< 0.0001
B	0.25	1	0.25	0.54	0.4848
C	64.75	1	64.75	140.47	< 0.0001
AB	1.21	1	1.21	2.63	0.1487
AC	25.44	1	25.44	55.18	0.0001
BC	0.17	1	0.17	0.36	0.5669
A^2	20.9	1	20.9	45.34	0.0003
B^2	42.3	1	42.3	91.76	< 0.0001
C^2	27.6	1	27.6	59.87	0.0001
Residual	3.23	7	0.46		
Lack of Fit	2.59	3	0.86	5.38	0.0689
Pure Error	0.64	4	0.16		
Total	263.81	16			

Table S2. Analytical model parameters for breaking elongation

Source	Sum of	df	Mean	F	P
Model	27.45	9	3.05	43.75	< 0.0001
A	6.04	1	6.04	86.68	< 0.0001

B	8.24E-04	1	8.24E-04	0.012	0.9165
C	9.34	1	9.34	133.98	< 0.0001
AB	0.078	1	0.078	1.13	0.324
AC	0.96	1	0.96	13.81	0.0075
BC	0.54	1	0.54	7.68	0.0276
A^2	2.45	1	2.45	35.12	0.0006
B^2	4.28	1	4.28	61.43	0.0001
C^2	2.65	1	2.65	37.94	0.0005
Residual	0.49	7	0.07		
Lack of Fit	0.28	3	0.093	1.78	0.2901
Pure Error	0.21	4	0.052		
Total	27.94	16			

Table S3. Analytical model parameters for transmittance

Source	Sum of	df	Mean	F	P
Model	141.18	9	15.69	50.58	< 0.0001
A	8.32	1	8.32	26.83	0.0013
B	0.24	1	0.24	0.77	0.4085
C	27.34	1	27.34	88.15	< 0.0001
AB	0.044	1	0.044	0.14	0.7187
AC	3.45	1	3.45	11.12	0.0125
BC	3.22	1	3.22	10.37	0.0146
A^2	9.89	1	9.89	31.89	0.0008
B^2	30.59	1	30.59	98.64	< 0.0001
C^2	46.57	1	46.57	150.17	< 0.0001
Residual	2.17	7	0.31		
Lack of Fit	1.34	3	0.45	2.13	0.2387
Pure Error	0.83	4	0.21		
Total	143.36	16			

Table S4. The standard curve regression equation and correlation coefficient of Cefradine

Medium	Regression equation	R^2
pH=1.2	A=0.01152c+0.0006	0.9998
pH=4.5	A=0.0209c+0.0015	0.9982
pH=6.8	A=0.0185c+0.0081	0.9989
pH=7.0	A=0.0150c+0.0023	0.9983

Table S5. The standard curve regression equation and correlation coefficient of Ranitidine

Medium	Regression equation	R^2
pH=1.2	A=0.0020c+0.0003	0.9963
pH=4.5	A=0.0506c+0.0055	0.9973
pH=6.8	A=0.0536c+0.0017	0.9999
pH=7.0	A=0.0493c+0.0038	0.9998

Supplementary Methods for Polymer Physicochemical Parameters

The physicochemical parameters of gellan gum (GG), ι-carrageenan (ι-C), and hydroxypropyl methylcellulose (HPMC) reported in this study were based on supplier specifications and quality documents. These parameters were not independently re-determined in our laboratory, because the focus of the present work was capsule formulation development and performance evaluation rather than re-characterization of the commercial raw materials. The corresponding standard methods used by the manufacturers are summarized below for reference.

(1) Molecular weight

The molecular-weight values of GG, ι-C, and HPMC were provided by the manufacturers. According to the supplier information, the average molecular weight was determined by gel permeation chromatography (GPC) equipped with a multi-angle laser light scattering (MALLS) detector.

(2) Sulfate content of ι-carrageenan

For ι-carrageenan, the **sulfate content** (as SO_4) was determined according to **GB 1886.169-2016**[1]. Briefly, 15 g of sample was accurately weighed (to 0.0002

g), dispersed in 500 mL isopropanol solution, gently stirred for 4 h, filtered, washed twice with isopropanol solution, and dried at 105 °C to constant weight. Then, 1 g of the pretreated sample was accurately weighed (to 0.0002 g), mixed with 50 mL hydrochloric acid solution, and refluxed for 1 h. Subsequently, 25 mL hydrogen peroxide solution was added and the mixture was further refluxed for approximately 5 h until the solution became completely clear. The resulting sample solution was transferred to a 600 mL beaker and heated to boiling, after which 10 mL barium chloride solution was added dropwise under continuous stirring. The mixture was maintained near boiling for about 2 h with a cover to allow complete precipitation. The precipitate was collected on quantitative filter paper, washed with hot water until chloride-free, transferred together with the filter paper into a pre-ignited constant-weight crucible, and ignited at 800 ± 25 °C to constant weight. After cooling in a desiccator, the sulfate content (as SO_4) was calculated gravimetrically from the mass of barium sulfate. The mass fraction w1 of sulfate ester (calculated as SO4) is calculated by Formula (1):

$$w1= \frac{m(m1\text{-}m2)\times 0.4116}{m}\times 100\% \qquad \text{(S1)}$$

Where:m1, Mass of the crucible plus residue, unit: gram (g); m2; Mass of the crucible, unit: gram (g);m Mass of the sample, unit: gram (g). The final result was expressed as the arithmetic mean of parallel determinations.

(3) Viscosity of ι-carrageenan

For ι-carrageenan, the **viscosity** was determined according to **GB 1886.169-**

2016[1]**.** Briefly, Weigh 7.5 g of carrageenan sample (accurate to 0.0002 g) into a pre-weighed 600 mL beaker, add approximately 450 mL of deionized water, and stir for 10-20 min to achieve complete dispersion. Add deionized water to adjust the total mass of the solution to 500 g. Heat the mixture in a water bath with continuous stirring, and terminate heating after 20-30 min when the temperature reaches 80℃. Compensate for evaporation loss with deionized water, cool the solution to 76-77℃, and equilibrate in a 75℃ constant-temperature water bath. Prior to measurement, preheat the spindle and guard of the rotational viscometer to 75℃ in water, dry thoroughly, and mount on the viscometer fitted with a No. 1 spindle (19 mm in diameter, 65 mm in length). The viscosity measurement was conducted at a rotational speed of 30 r/min.

(4) Hydroxypropoxy content of HPMC

For HPMC, the **content of hydroxypropoxy group** was determined according to **GB 1886.109-2015**[2]. Briefly, 2.5 g of toluene was diluted to 1000 mL with o-xylene to prepare the internal standard solution. Adipic acid, hydroiodic acid, and internal standard solution were mixed to prepare the standard solution. The sample was reacted with adipic acid, internal standard solution, and hydroiodic acid at 150℃ for 60 min, cooled, and the upper layer was taken. Gas chromatographic analysis was performed on a 1.8 m × 4 mm column (125-150 um siliceous earth packed with 10% methyl silicone oil) at 100℃, with helium as carrier gas (20 mL/min) and 2 μL injection volume. The hydroxypropoxy content was calculated via peak area ratio and response factor.

(5) Viscosity of HPMC

According to **GB 1886.109-2015**[2], the **viscosity** of HPMC was measured by dispersing an appropriate amount of sample equivalent to 2 g of dried solid in 98 g of water at 80-90°C in a 250 mL wide-mouth centrifuge bottle. After mechanical stirring for 10 min, the bottle was placed in an ice bath to equilibrate the solution temperature, and the total mass was adjusted to 100 g if necessary. After centrifugation to remove entrained air, the solution temperature was adjusted to 20 ± 1°C, and the viscosity was determined using a viscometer.

References:

[62] National Health and Family Planning Commission of the People's Republic of China. GB 1886.169-2016 National Food Safety Standard: Food Additive Carrageenan [S]. Beijing: China Standards Press, 2016.

[63] National Health and Family Planning Commission of the People's Republic of China. GB 1886.109-2015 National Food Safety Standard: Food Additive Hydroxypropyl Methyl Cellulose (HPMC) [S]. Beijing: China Standards Press, 2015.
